# Dictionary Holder Noyes'

**Published:** 1882-02

**Authors:** 


*Dictionary Holders.*—Experience in the use of one of L. W. Noyes' Dictionary Holders enables us to recommend it as one of the most convenient and attractive appliances to facilitate reference that has been offered to the public. By its use we are enabled to refer to the contents of all large works difficult from their size to handle, without the exercise of considerable strength and inconvenience. It is certainly a valuable addition to all libraries, and greatly facilitates investigation and research, and can be obtained from L. W. Noyes, 99 & 101 West Monroe St., Chicago, Ill.

